# Consequences of cyberbullying behaviour in working life: The mediating roles of social support and social organisational climate

**DOI:** 10.1108/IJWHM-10-2016-0075

**Published:** 2017-10-02

**Authors:** Tuija Muhonen, Sandra Jönsson, Martin Bäckström

**Affiliations:** 1 Centre for Work Life and Evaluation Studies, Malmö University, Malmö, Sweden; 2 Department of Urban Studies, Faculty of Culture and Society, Malmö University, Malmö, Sweden; 3 Department of Psychology, Lund University, Lund, Sweden

**Keywords:** Social support, Social organizational climate, Cyberbullying behaviour, Work life

## Abstract

**Purpose:**

The purpose of this paper is to explore health- and work-related outcomes of cyberbullying behaviour and the potential mediating role of social organisational climate, social support from colleagues and social support from superiors.

**Design/methodology/approach:**

Altogether 3,371 respondents participated in a questionnaire study.

**Findings:**

The results of this study indicate that social organisational climate can have a mediating role in the relationship between cyberbullying behaviour and health, well-being, work engagement and intention to quit. Contrary to earlier face-to-face bullying research, the current study showed that cyberbullying behaviour had stronger indirect than direct relationships to health, well-being, work engagement and intention to quit.

**Practical implications:**

Communication through digital devices in work life is becoming more prevalent, which in turn increases the risk for cyberbullying behaviour. Organisations need therefore to develop occupational health and safety policies concerning the use of digital communication and social media in order to prevent cyberbullying behaviour and its negative consequences.

**Originality/value:**

Cyberbullying behaviour among working adults is a relatively unexplored phenomenon and therefore this study makes valuable contribution to the research field.

Through the increasing use of digital media such as e-mails, text messages and social network sites, new forms of harassing behaviour in workplaces have emerged, referred to as cyberbullying or cyber-harassment (Borstorff *et al.*, 2007; Smith *et al.*, 2008). Research on cyberbullying and its consequences has mainly been conducted among adolescents and schoolchildren (Slonje and Smith, 2008; Dooley *et al.*, 2009), while the phenomenon among adults in working life has only recently started to attract researchers’ attention (Brack and Caltabiano, 2014; Farley *et al.*, 2015; Forssell, 2016; Privitera and Campbell, 2009). Being a relatively new phenomenon, the pioneering studies investigating cyberbullying in working life have mainly focussed on determining its prevalence (Baruch, 2005; Brack and Caltabiano, 2014; Privitera and Campbell, 2009; Farley *et al.*, 2015; Forssell, 2016). These studies show varying prevalence rates from 9 to 21 per cent, but as in face-to-face bullying research the variation can be due to different ways of measuring and defining bullying. Since there is a paucity of research investigating cyberbullying in working life we refer to studies focussing on cyberbullying among youngsters/adolescents, as well studies on face-to-face bullying in work life.

There is an ongoing discussion among bullying researchers concerning what characterises cyberbullying compared to face-to-face bullying (Olweus, 2013; Runions *et al.*, 2013; Slonje and Smith, 2008). When it comes to face-to-face bullying, most researchers agree that the negative acts are conducted repeatedly and systematically over time, and that there is a power imbalance, i.e. the individuals who are exposed to the negative acts have difficulties defending themselves (Einarsen *et al.*, 2011). In a similar way, cyberbullying among youngsters has been defined as an aggressive intentional act carried out by a group or individual, using electronic forms of contact, repeatedly and over time against a victim who cannot easily defend him or herself (Smith *et al.*, 2008, p. 376). However, repetition of negative acts and power imbalance can have a different meaning in cyberbullying compared with face-to-face bullying (Dooley *et al.*, 2009; Runions *et al.*, 2013; Wang *et al.*, 2011).

Cyberbullying behaviours have a large potential audience as they are communicated through different digital media, which means that they can be easily stored, shared and viewed repeatedly. Therefore, the importance or meaning of repetition can be discussed as one act, e.g. sending a message or uploading a photo in social media can be viewed several times and easily distributed (Patchin and Hinduja, 2006; Slonje and Smith, 2008). From the targets’ perspective every hit on the webpage where a picture or video clip is uploaded could be counted as repetition (Slonje and Smith, 2008). The concept of power imbalance is more complex in the cyberbullying context, as the formal power position can be challenged, e.g. by the invisibility of the actions and the perpetrators technological knowledge (Dooley *et al.*, 2009).

Other special characteristics of cyberbullying include high accessibility and to some extent even anonymity of the perpetrators (Kowalski *et al.*, 2014; Slonje and Smith, 2008). High accessibility implies that since we are more or less continuously connected to the internet, the victims of cyberbullying behaviour have few possibilities to escape the harassing behaviour (Patchin and Hinduja, 2006; Slonje and Smith, 2008; Tokunaga, 2010). The victims can be targeted whenever and wherever, even outside the workplace and after office hours. The harassment can continue, or even begin, when the workday is actually over. Thus, it is very difficult for the victims to avoid cyberbullying behaviour, which can result in feelings of powerlessness and power imbalance over time (Dooley *et al.*, 2009; Lutgen-Sandvik *et al.*, 2007).

As cyberbullying in working life is a relatively new phenomenon its consequences remain yet to be studied. Building upon earlier face-to-face bullying research based on occupational stress theory (cf. Hogh *et al.*, 2011), the aim of this study was to analyse how cyberbullying behaviour in working life was related to the following outcome variables: health, intention to quit, psychological well-being and work engagement. A further aim was to investigate whether social organisational climate, social support from colleagues and superior mediate the relationship between cyberbullying behaviour and the outcome variables.

## Health-related outcomes

Traumatic events such as bullying can have devastating effects on the victim’s health and well-being (Hogh *et al.*, 2011). Earlier research has shown that victims of workplace face-to-face bullying report more health problems, more depressive symptoms, psychological stress, poorer well-being and are absent from work more often, compared with other employees (Dehue *et al.*, 2012; O’Driscoll *et al.*, 2011; Moayed *et al.*, 2006; Nielsen *et al.*, 2012; Vie *et al.*, 2010). These results are corroborated by a meta-analytic review (Nielsen and Einarsen, 2012) showing that exposure to bullying was associated with health- and well-being-related outcomes, such as mental and physical health problems, symptoms of post-traumatic stress and burnout. There are also indications that the negative health consequences of workplace bullying such as sick-listing, poor self-rated health and depressive symptoms may persist for a long time, even when the bullying no longer continues (Bonde *et al.*, 2016).

## Work-related outcomes

Earlier studies of face-to-face workplace bullying have shown that besides health and well-being, bullying is also related to several work-related outcomes such as lower job satisfaction, negative work attitudes and lower perceived job performance (Hoel *et al.*, 2011; O’Driscoll *et al.*, 2011). Further, bullying victims report lower work engagement and organisational commitment than non-victims (Nielsen and Einarsen, 2012). Several studies have shown that workplace bullying is related to higher intention to leave the organisation (Djurkovic *et al.*, 2004; Nielsen and Einarsen, 2012). It is understandable that the victims want to leave a workplace where they are bullied and, this is also what bullying victims often are advised to do in order to get away from a strenuous situation (Zapf and Gross, 2001).

Thus, several studies have indicated that face-to-face bullying has negative consequences for victims’ health and well-being (Dehue *et al.*, 2012; O’Driscoll *et al.*, 2011; Moayed *et al.*, 2006; Nielsen *et al.*, 2012; Vie *et al.*, 2010). Further, there are indications that face-to-face bullying also results in lower work engagement and higher intention to leave their job (Djurkovic *et al.*, 2004; Nielsen and Einarsen, 2012). In line with these results from face-to-face workplace bullying, it could be expected that cyberbullying behaviour has similar consequences. We therefore hypothesise that:

H1. Increasing cyberbullying behaviour is related to poorer health, higher intention to quit, lower well-being and lower work engagement.

## Mediating factors

In this study we view cyberbullying behaviour as a stress factor that can lead to different negative outcomes for the targeted individuals (Hauge *et al.*, 2010; Rodríguez-Muñoz *et al.*, 2009). Besides direct effects, cyberbullying behaviour can also have indirect effects on the outcome variables. Consequently, it is important to investigate factors that mediate in the relationship between cyberbullying and negative outcomes related to health and work. Social support from superiors and social support from colleagues are examples of these kinds of factors (Cassidy *et al.*, 2014; Zapf *et al.*, 1996).

### Support from colleagues and superiors

Employees that have been exposed to bullying often report low support from their superiors (Hansen *et al.*, 2006; Zapf *et al.*, 1996), and as several authors have pointed out, the superiors can even be the perpetrators (Tepper, 2007; Vandekerckhove and Commers, 2003; Zapf *et al.*, 2011). Victims are often left with feelings of being ignored, minimised and not being believed when raising their concerns with the superior or human resources department (Keashly, 2001; Lewis and Orford, 2005). Also, support from colleagues is often rated as low by bullying targets (Zapf *et al.*, 1996). On the other hand, several studies indicate that support from colleagues can reduce the negative impact of bullying (Djurkovic *et al.*, 2004), e.g. perceived organisational support has been found to moderate the relationship between workplace bullying and victims’ intention to leave (Djurkovic *et al.*, 2008).

Thus, as previous research has indicated that social support from colleagues and social support from superiors can be important factors in relation to the victims of face-to-face workplace bullying, we hypothesise that:

H2a. Social support from colleagues mediates the relationship between cyberbullying behaviour and the outcomes: health, intention to quit, well-being and work engagement.

H2b. Social support from superiors mediates the relationship between cyberbullying behaviour and the outcomes: health, intention to quit, well-being and work engagement.

### Social organisational climate

Earlier studies have pointed out the mediating role of social climate in the organisation in relation to employees’ well-being, and even for their possibilities for learning and development at the workplace (Muhonen *et al.*, 2013; Jönsson *et al.*, 2015). Organisational culture and climate has also been shown to play an important role when it comes to face-to-face bullying (Powell *et al.*, 2015; Vartia, 1996). A negative organisational climate and culture can be directly related to face-to-face bullying (Bowling and Beehr, 2006), or indirectly related through a stressful or competitive work environment, which triggers face-to-face bullying (Aquino and Lamertz, 2004; Salin, 2003). Workplace bullying has earlier been regarded as a consequence of organisational climate factors (Bowling and Beehr, 2006; Einarsen *et al.*, 2011), but this causality has been questioned (Hauge *et al.*, 2010). Giorgi (2012) maintains that workplace bullying can be a cause rather than a consequence of organisational climate. Furthermore, according to Giorgi (2012), workplace bullying can have an indirect effect, e.g. on health via organisational climate. As earlier face-to-face bullying research indicates that organisational climate can have an indirect mediating effect in the relationship between workplace bullying and different outcomes, we hypothesise that:

H3. Social organisational climate mediates the relationship between cyberbullying behaviour and the outcomes: health, intention to quit, well-being and work engagement.

Furthermore, it can be postulated that cyberbullying may affect how social support from superior and social support from colleagues are experienced and that this in turn can have a detrimental effect on the experienced social organisational climate and thereby the different outcomes. In other words, if the bullying victims perceive less social support from superior and from colleagues, this could explain the relation to the perception of social organisational climate. In our model social climate can mediate the relation between support and outcomes, and therefore hide a possible influence of support from cyberbullying through support from superior and from colleagues.

Thus, we formulated following hypotheses:

H4. Social support from colleagues, through its influence on the social climate mediates the relationship between cyberbullying behaviour and outcomes: health, intention to quit, well-being and work engagement.

H5. Social support from superior, through its influence on the social climate mediates the relationship between cyberbullying behaviour and outcomes: health, intention to quit, well-being and work engagement.

## Method

### Participants and procedure

Data were collected between 17 April and 20 May 2014 by TNS Sifo, a public opinion poll and market research company. TNS Sifo has an online web panel consisting of a nationally representative random sample of 140,000 people aged 16 years or older. The questionnaire was distributed to a sample of individuals aged between 25 and 65 who were resident in Southern Sweden.

In total, 3,885 individuals responded the questionnaire, but as the aim of the study was to study cyberbullying in working life, those individuals who had not been employed during the last six months (*n*=514) were excluded from the study. The total number of participants was therefore 3,371, giving a response rate of 42 per cent.

Of the participants, 49 per cent were women, 32 per cent had a managerial position and the mean age was 50 years (SD=9.63). A majority (60 per cent) had university education and were working full-time (82 per cent). Also, a majority (73 per cent) reported that they used digital tools (computer, mobile phone, iPad, etc.) very often or always at their work.

### Measures

#### Demographics

The demographic questions included age, gender, educational level (1=university degree or 2=not), organisational level (1=managerial position or 2=not).

#### Cyberbullying behaviour

Cyberbullying behaviour was measured by a short version of Cyberbullying Behaviour Questionnaire (CBQ-S) developed by Jönsson *et al.* (2017). The reliability and validity of the questionnaire is reported and discussed in Jönsson *et al.* (2017). The CBQ-S consists of seven items; a sample item is: “Your work performance has been commented in negative terms on the internet” (*α*=0.88). Before they responded the following instruction was given to the participants “The following behaviours are often seen as examples of negative behaviour in the workplace that may occur via the use of technology. When responding consider every act in relation to these eight types of technologies: text messaging; pictures/photos or video clips, phone calls; e-mail; chat rooms; instant messaging; websites; and social networking websites (e.g. Facebook, Twitter, YouTube). Over the last six months, how often have you been subjected to the following negative acts related to your work through different forms of technology?”

The respondents rated the items on a five-point scale, 1=never, 2=now and then, 3=monthly, 4=weekly, 5=daily, in line with earlier bullying research (Einarsen *et al.*, 2009; Sprigg *et al.*, 2012). As the scale was very skewed the three highest rating categories (3-5) were collapsed into one and the whole rating scale was moved by one point for a new range from 0 to 2. This is a common procedure in several bullying studies (Einarsen *et al.*, 2009; Cassidy *et al.*, 2014), as the phenomenon assessed does not have a normal statistical distribution.

#### Social organisational climate

Social organisational climate was assessed by five items from QPSNordic (Dallner *et al.*, 2000). “What is the climate like in your work unit: (1) Competitive (2) Encouraging and supportive, (3) Distrustful and suspicious (4) Relaxed and comfortable (5) Rigid and rule-based?” The participants responded using a five-point scale ranging from 1=very little/not at all to 5=very much. Cronbach’s *α* was 0.78.

#### Social support from superior and support from colleagues

Social support from superior was measured by two items from COPSOQ II (Pejtersen *et al.*, 2010; Berthelsen *et al.*, 2014). An example item is “How often is your nearest superior willing to listen to your problems at work?” Cronbach’s *α* was 0.90.

Social support from colleagues was also measured by two items from COPSOQ II. A sample item is “How often do you get help and support from your colleagues?” Cronbach’s *α* was 0.93. All the support items were rated on a five-point scale ranging from 1=very often to 5=very rarely. Before analyses the original values were reversed so that higher values indicate increasing support.

#### Psychological well-being

Well-being was assessed by General Health Questionnaire-12, originally developed by Goldberg (1972); the Swedish version was developed by Sconfienza (1998). The scale consists of 12 items and a sample item is: “I have been able to face up my problems”. The respondents rated the items on a four-point scale from 1 (=disagree very much) to 4 (=agree very much). Cronbach’s *α* was 0.76. In the models we reduced the number of variables to six by creating random parcels.

#### Work engagement

Work engagement was assessed by a short form of the Utrecht Work Engagement Scale (UWES-9; Schaufeli *et al.*, 2006). The scale consists of nine items and an example is: “I am enthusiastic about my job”. The responses were rated on seven-point scale from 1 (=never) to 7 (=always). Cronbach’s *α* was 0.95. In the models we reduced the number of variables to four by creating random parcels.

#### Intention to quit

Intention to quit was measured by one item: “How often do you consider looking for work somewhere else?” (Pejtersen *et al.*, 2010; Berthelsen *et al.*, 2014). Ratings were made on a five-point scale from 1 (=very seldom) to 5 (=very often).

#### Health

Health was assessed by one item: “In general, would you say that your health is 1) Excellent, 2) Very good, 3) Good, 4) Not so good, 5) Poor?” Before analyses the original values were reversed so that higher values indicate better health. According to Lindberg *et al.* (2009), this single question has proven to be able to predict future sick leave and can therefore be used for identifying risk groups that are in need of preventive measures.

### Statistical analysis

**Figure 1:**
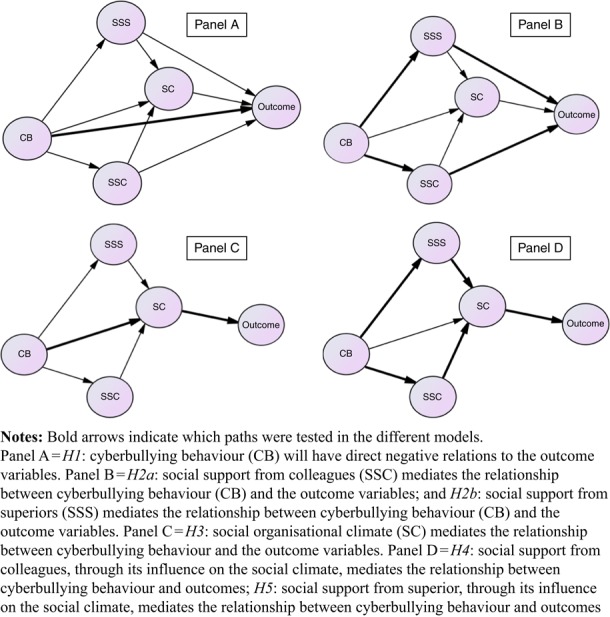
The models tested in this study

The data were initially analysed by descriptive statistics, we report means, standard deviations and the correlations between the variables. Based on our hypotheses, we developed models shown in Figure 1 that were estimated with MPlus 7.1 (Muthén and Muthén, 1998/2012).

#### Random parcels

Some of the scales used to create the latent variables of the models consisted of many items. To increase the reliability and validity of the observed variables in models, and to not have models that do not fit because of covariance between items on lower level, or collinearity between groups of items, for some of the scales, we created small random parcels, consisting of two or three items. Since all inventories had rather good homogeneity this should not influence the conclusions.

#### Measurement models

The tested models included six latent variables of which four consisted of more than two observed variables. These four latent variables were tested separately to investigate whether their measurement models fitted the data. Table I displays the standardized loadings and the comparative fit indexes (CFI) of the measurement models. All models fitted data well with CFI on or above 0.950.

The starting model includes all paths and the hypotheses were tested by investigating to what extent the measurement fit was decreased when paths were deleted (set to zero). The mediation hypotheses were tested by investigating the indirect effects in the models (see Figure 1, which shows the paths tested and the models used to test the paths). Cyberbullying is the sole exogenous independent variable in the model and its influence on the outcome variables work engagement, health, intention to leave and well-being are supposed to be indirect through social support from superior, social support from colleagues and social organisational climate. In the starting model there are also direct effects on the outcome variables. The influence of support from superior and support from colleagues is also supposed to influence the dependent variables through social organisational climate, but direct effects were included in the starting model. All outcome variables were defined to be correlated, and in addition, the two support latent variables were free to correlate. The models were estimated with the MPlus program with the observed variables of cyberbullying, social organisational climate, intention to quit and health defined as categorical. The weighted least square estimator (WLSMV) was used in all estimations. The *α* was set at 0.01 since the sample was very large.

## Results

### Descriptive statistics

The descriptive statistics for the observed variables and correlations for the latent variables that were included in the model are shown in Table II. When it comes to the prevalence of cyberbullying the study showed that 9.7 per cent of the respondents could be labelled as cyberbullied in accordance with Leymann’s (1996) cut-off criterion, i.e. exposure to at least one cyberbullying act weekly during the last six months. This finding is reported and discussed in Forssell (2016).

### The direct and indirect relationships of the study variables

The first hypothesis (*H1*) stated that increasing cyberbullying behaviour would be related to poorer health, higher intention to quit, lower well-being and lower work engagement (see Figure 1, panel 1A). In general, the relations between cyberbullying behaviour and the outcome variables were weak and the only significant one, work engagement, was found to be in the opposite direction (see Table III, Model 1). Based on the low correlations between cyberbullying behaviour and the outcome variables: health, intention to quit, psychological well-being and work engagement, direct relations were not supported, and hence the hypothesis (*H1*) was rejected. In the next model, we tested if indirect relations between cyberbullying behaviour and the outcome variables were more important.

The second hypothesis (Figure 1, panel 1B) tested if social support from colleagues (*H2a*) and social support from superior (*H2b*) could mediate the relationship between cyberbullying behaviour and the outcome variables (health, intention to quit, well-being and work engagement). The results showed that only work engagement had a direct relation to social support from superior (see Table III, Model 2); it was found that cyberbullying behaviour was indirectly related to work engagement over support from superior (*β*=–0.027, *p*=0.001). In general, however, there was no support that social support from colleagues (*H2a*) or social support from superior (*H2b*) mediate the relationship between cyberbullying behaviour and the outcome variables.

The third hypothesis (see Figure 1, panel 1C) tested whether social organisational climate could mediate the relationship between cyberbullying behaviour and the outcome variables (health, intention to quit, well-being and work engagement). The analyses showed that all four indirect relations were significant (see Table III, Model 3, and Table IV). This provides strong support for mediating relation (*H3*), suggesting that cyberbullying behaviour can have a negative influence on the social organisational climate and that this in turn can affect the outcome variables.

The fourth and fifth hypotheses (see Figure 1, panel 1D) tested, whether social support from colleagues and superior, through its influence on the social climate, mediates the relationship between cyberbullying behaviour and outcomes. These were tests of the significance of the indirect relations part of Model 3 shown in Table III. It was found that the indirect relation between cyberbullying behaviour and the outcome variables in general was stronger for social support from superiors than for social support from colleagues, but all indirect effects were significant when they also went through social organisational climate (see Table IV). This verifies the hypotheses that social support from colleagues and social support from superiors mediate the effects of cyberbullying behaviour on the outcome variables. However, social support from superiors and social support from colleagues also influence the social organisational climate and this indirect relation appears to be the most important explanation for how cyberbullying behaviour can influence the outcome variables.

## Discussion

Cyberbullying behaviour among working adults is a relatively new and unexplored phenomenon (Brack and Caltabiano, 2014; Farley *et al.*, 2015), and therefore the current study makes a unique and timely contribution to the research field. Based on earlier studies of face-to-face bullying the purpose of the current study was to analyse how cyberbullying behaviour in working life was related to health, intention to quit, psychological well-being and work engagement. Further, the potential mediating relations of social organisational climate, social support from colleagues and superior between cyberbullying behaviour and the outcome variables were explored with SEM analyses.

The results showed that cyberbullying behaviour had only one significant direct relation to the outcome variables, namely, positive relationship to work engagement. Therefore, the first hypothesis (*H1*) postulating that increasing cyberbullying behaviour would be directly related to poorer health, higher intention to quit, lower well-being and lower work engagement was not supported. These findings suggest that cyberbullying behaviour does not have a direct and unique influence on intention to quit, well-being and work engagement (Dehue *et al.*, 2012; O’Driscoll *et al.*, 2011; Nielsen and Einarsen, 2012).

Likewise, the results did not support the hypotheses postulating that social support from colleagues (*H2a*) and social support from superior (*H2b*) would mediate between cyberbullying behaviour and the outcome variables (health, intention to quit, psychological well-being and work engagement). These findings differ from studies of face-to-face bullying pointing out colleagues’ and supervisors’ crucial role in the bullying situations (Djurkovic *et al.*, 2004; Hansen *et al.*, 2006; Zapf *et al.*, 2011).

When it comes to social organisational climate the results confirmed the hypothesis (*H3*) by showing a mediating relationship between cyberbullying behaviour and the outcome variables (health, intention to quit, psychological well-being and work engagement). This implies that cyberbullying behaviour may influence the experience of the social organisational climate and thereby can have negative consequences on health, well-being, engagement and intention to quit. This result is in line with earlier research of face-to-face bullying (Powell *et al.*, 2015; Vartia, 1996).

Further, the results supported the hypotheses that the social support from colleagues, and social support from superior mediates the relation between cyberbullying behaviour and the outcome variables through social organisational climate (*H4* and *H5*). The indirect relation between cyberbullying behaviour and the outcome variables was in general stronger for social support from superiors than for social support from colleagues, but all indirect effects were significant when they also went through social organisational climate. The results indicate that social support from superiors and social support from colleagues can influence the social organisational climate, and that this indirect relation is the most important explanation for how cyberbullying behaviour can influence the outcome variables.

In sum, the results showed both some similarities and some differences compared with face-to-face workplace bullying research. Rather than having a direct relationship with the outcome variables: health, intention to quit, psychological well-being and work engagement (Nielsen and Einarsen, 2012; O’Driscoll *et al.*, 2011), cyberbullying behaviour had stronger indirect relationships to the outcome variables through the other studied variables. Especially, social organisational climate seems to play a mediating role between cyberbullying behaviour and the outcome variables: health, intention to quit, psychological well-being and work engagement. The study shows that cyberbullying behaviour can influence both the amount of social support experienced from superiors and colleagues, and more generally can influence the social organisational climate. Another way of formulating this is that health-related (health and well-being) and work-related (intention to quit, work engagement) consequences of cyberbullying behaviour in working life, in general seem to be mediated by the experience of the social organisational climate, social support from colleagues and superior.

Further, it appeared that social support from superior had a somewhat stronger relationship than support from colleagues to the outcome variables. This is in accordance with face-to-face bullying studies (Djurkovic *et al.*, 2008), and can also have practical implications when dealing with cyberbullying behaviour.

The study has a limitation due to its cross-sectional design, which prevents us from making causal inferences. Earlier research (cf. Zapf, 1999) has also pointed out that there is a general problem in bullying research concerning the differentiation between causes and consequences of bullying behaviour. For example, a study by Rodríguez-Muñoz *et al.* (2009) showed that negative job-related well-being was the cause rather than a consequence of workplace face-to-face bullying. Likewise, negative working conditions can be either cause or a consequence of workplace bullying (Zapf, 1999). Stressful work environment can produce behavioural and affective reactions in certain individuals that in turn increase the risk of them being victimized (Bowling and Beehr, 2006). We have chosen to use the term consequences in this study, even though we are not claiming to prove causality between the study variables. The relationships between cyberbullying behaviour, social organisational climate and the outcome variables can be dynamic rather than unidirectional. Therefore, it can be discussed whether cyberbullying behaviour is caused by poor social organisational climate or the other way round, or if the relationship is reciprocal. In order to address the causality experimental and/or longitudinal design should be applied in future studies.

Another limitation is that some of the scales were measured with few items and both intention to leave and health were measured by only one item. However, earlier studies (Lindberg *et al.*, 2009; Tham, 2007) have indicated that these measures have good predictive validity. Further, the technological development and increasing digitalisation can give rise to new forms of cyberbullying behaviour that might not be assessed by the questionnaire used in this study.

The current study was conducted in Sweden where the prevalence of workplace bullying, like in the other Scandinavian countries, is lower compared with other European and non-European countries (Nielsen *et al.*, 2010; Forssell, 2016). As cyberbullying research is in its early stages yet there is need for further research in Sweden but also in other countries in order to contextualise the findings of the current study.

## Conclusion

The results of this study indicate that social organisational climate can have a mediating role in the relationship between cyberbullying behaviour and health, well-being, work engagement and intention to quit. Contrary to earlier face-to-face bullying research, the current study showed that cyberbullying behaviour had stronger indirect rather than direct relationships to health, well-being, work engagement and intention to quit. As communication through digital devices and social media in work life increases, there is a danger that cyberbullying behaviour also might become more prevalent. Organisations need therefore to develop organisational health and safety policies concerning the use of digital communication and social media, as well as activities in order to prevent cyberbullying behaviour and its negative effects. As this study was conducted in the Swedish context, there is need for research in other countries concerning cyberbullying behaviour in working life, its causes and consequences, but also to develop evidence-based interventions.

## Figures and Tables

**Table I:** Standardised loadings and CFI

	Estimations							
Measurement models	Item 1	Item 2	Item 3	Item 4	Item 5	Item 6	Item 7	CFI
Cyberbullying	0.685	0.738	0.821	0.655	0.762	0.747	0.780	0.958
Work engagement	0.915	0.955	0.921	0.860				0.984
Social climate	0.541	0.760	0.749	0.843	0.600			0.950
Well-being	0.761	0.617	0.639	0.761	0.741	0.694		0.959

**Table II:** Descriptives and correlations between the latent variables included in the model

	1	2	3	4	5	6	7	8
1. Cyberbullying								
2. Social support superior	–0.25							
3. Social support colleagues	–0.19	0.60						
4. Social climate	–0.25	0.45	0.40					
5. Work engagement	–0.11	0.18	0.17	0.24				
6. Well-being	–0.14	0.37	0.30	0.44	0.34			
7. Health	–0.09	0.16	0.16	0.22	0.26	0.39		
8. Intention to quit	0.26	0.43	–0.29	–0.53	–0.31	–0.69	–0.25	
*M* (of scales)	0.95	8.02	8.67	18.75	46.86	39.94	2.35	2.30
SD (of scales)	1.52	2.06	1.67	3.77	9.20	5.33	0.92	1.24

**Notes:** Health and intention to quit are observed variables. All the correlations were significant, *p*<0.01

**Table III:** Model results and *β*-coefficients from structural models

	Standardized coefficients	Dependent variables						
Model	Independent variables	Social support superior	Social support colleagues	Social climate	Engagement	Well-being	Health	Intention to quit
Model 1	Cyberbullying	–0.361**	–0.277**	–0.247*	0.077*	–0.057	–0.009	0.058
*χ*^2^ (326): 1,950.3	Social support superior			0.358*	0.119**	0.012	0.014	–0.148**
CFI: 0.972	Social support colleagues			0.216*	–0.031	0.057	0.018	0.122**
RMSEA: 0.04	Social climate				0.501**	0.505**	0.251**	–0.479**
Model 2	Cyberbullying	–0.365**	–0.280**	–0.246**	0.079*			
*χ*^2^ (333): 1,902.5	Social support superior			0.366**	0.080*			–0.153**
CFI: 0.974	Social support colleagues			0.222**				0.137**
RMSEA: 0.039	Social climate				0.510**	0.561**	0.282**	–0.521**
Model 3	Cyberbullying	–0.359**	–0.281**	–0.273**	0.081*			
*χ*^2^ (336): 1,886	Social support superior			0.377**	0.077*			
CFI: 0.973	Social support colleagues			0.204**				
RMSEA: 0.039	Social climate				0.513**	0.591**	0.281**	–0.542**

**Notes:** *p<0.01; **p<0.001

**Table IV:** Total effect, direct effects and indirect effects

Effect	Health	Intention to quit	Well-being	Engagement
Total effects	–0.116**	0.276**	–0.248**	–0.176**
Direct from CB		0.065		0.095**
Indirect SocClim	–0.077**	0.148**	–0.161**	–0.143**
Indirect SupSup and SocClim	–0.038**	0.073**	–0.080**	–0.071**
Indirect SupCol and SocClim	–0.016**	0.031**	–0.034**	–0.030**

**Notes:** CB, cyberbullying behaviour; SocClim, social climate; SupSup, social support from superior; SupCol, social support from colleagues. **p<0.001
